# Rapid Cycle Deliberate Practice in Healthcare Simulation: a Scoping Review

**DOI:** 10.1007/s40670-021-01446-0

**Published:** 2021-11-02

**Authors:** Carly Ng, Nadia Primiani, Ani Orchanian-Cheff

**Affiliations:** 1 Division of Emergency Medicine, Department of Family and Community Medicine, University of Toronto, 200 Elizabeth Street, Toronto, ON M5G 2C4 Canada; 2 Library and Information Services, University Health Network, Toronto, ON Canada

**Keywords:** Rapid cycle deliberate practice, Simulation, Medical education, Scoping review

## Abstract

Rapid cycle deliberate practice (RCDP) is a type of simulation-based medical education (SBME) where learners cycle between deliberate practice and directed feedback until skill mastery is achieved before progressing to subsequent learning objectives. This scoping review examines and summarizes the literature on RCDP, compares RCDP to other modes of instruction, and identifies knowledge gaps for future research. Of the 1224 articles identified, 23 studies met inclusion criteria. The studies varied in design, RCDP technique implementation strategies, and outcome measures. RCDP is associated with positive outcomes in immediate learner performance. It is unclear if RCDP is superior to traditional simulation.

## Introduction

Simulation-based medical education (SBME) has been shown to improve knowledge, clinical skills, and behaviors when compared to traditional medical education [1]. It is also associated with improved downstream effects on patient care, patient outcomes, and reduced healthcare costs [2]. The most common or traditional method of simulation debriefing is facilitator-guided post-event debriefing; it occurs after a simulation scenario and is effective for achieving learning goals and understanding correct and incorrect actions [3, 4]. Within-event debriefing involves interrupting the simulation scenario for short and focused debriefing events to allow for coaching in real time [3]. There is limited evidence that one method of simulation instruction is better than another.

Rapid cycle deliberate practice (RCDP), introduced by Hunt et al. in 2014, is a type of within event debriefing that “rapidly cycle(s) between deliberate practice and directed feedback until skill mastery is achieved” [5]. RCDP builds on the concepts of deliberate practice and mastery learning. Deliberate practice, commonly used to gain expertise in music and sports, allows for repeated opportunities to practice a skill with directed feedback until learning objectives are met [6]. Mastery learning, which is associated with clinical skills retention and patient care improvement, consists of clear learning objectives, formative assessments, advancement to the next educational objective once mastery has been achieved, and continued practice [2]. In RCDP, learners meet predefined learning objectives before progressing to more challenging goals or scenarios that build upon previously mastered skills [5]. Hunt et al. describes three principles for RCDP [5]:

1. Apply the concepts of overlearning and automatization to create muscle memory by providing multiple opportunities for repetitive practice to “do it right.”
2. Provide expert or evidence-based feedback efficiently.
3. Foster an environment for psychological safety to allow learners to enthusiastically welcome the feedback.

Hunt et al*.* found that pediatric resuscitation skills and critical care performance improved with RCDP simulation [5].

A systematic review was published in 2017 of RCDP that consisted of 15 resources, only two of which were published studies [7]. As such, it became a narrative review that reported implementation strategies and outcome measures. Results were limited and inconsistent, and the authors recommended conducting a similar review in 2-years time as more material would be available due to the rapid emergence of RCDP [7]. Given the limited results of the last review and the relatively new method of RCDP, the goal of this scoping review is to examine the current RCDP literature to help guide future research and identify gaps in RCDP and simulation. Ultimately, this may help shape the future of simulation instruction in healthcare.

The objectives of this scoping review are to examine and summarize the literature of RCDP in SBME, including outcome measures and implementation strategies; assess if RCDP leads to improved clinical performance of healthcare professionals and healthcare trainees when compared to other modes of instruction; and identify knowledge gaps and opportunities for future RCDP research.

## Methods

A comprehensive search strategy was developed by a medical librarian to identify articles on rapid cycle deliberate practice (RCDP). The initial search strategy was developed for Ovid Medline and adapted from the one used by Taras and Everett using a combination of text words [7]. The search strategy was then customized for each database. Searches of the following databases were executed on October 13, 2020: Ovid MEDLINE ALL, Ovid EMBASE, Cochrane Database of Systematic Reviews (Ovid), Cochrane Central Register of Controlled Trials (Ovid), PsycINFO (Ovid), and CINAHL with Full Text (EBSCO). There were no restrictions on publication period. Limits were imposed for English language. No other limits were applied. See Appendix I for database search strategies. Additional search methods included screening reference lists of eligible studies.

The inclusion criteria were based on the PICO (population, intervention, comparison, outcome) approach. The target population was healthcare professionals and trainees. The intervention was RCDP healthcare simulation. The comparison was traditional simulation, other instruction, or no comparison. The outcomes examined were learner satisfaction, knowledge acquisition, clinical performance, implementation of skills in practice, quality of care, and patient outcomes. English language full-text published studies of any design were included.

**Fig. 1:**
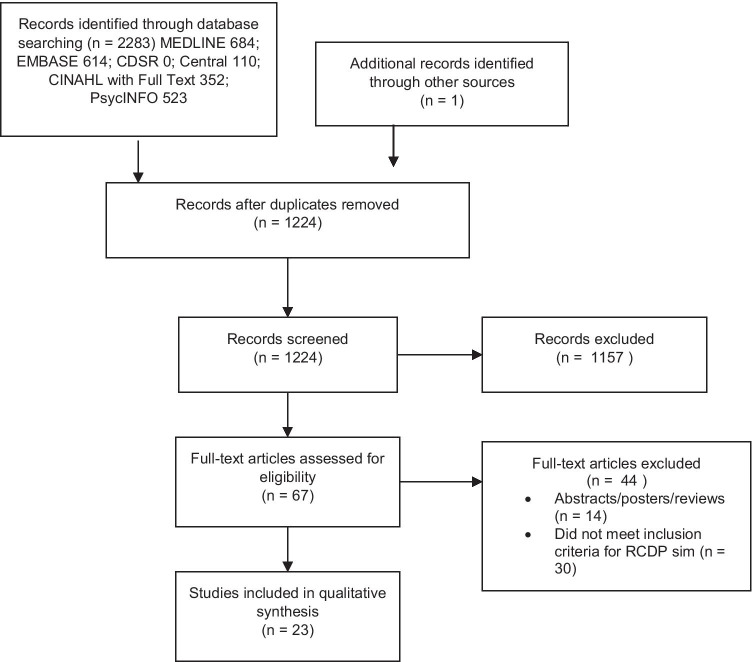
PRISMA flow diagram

The initial database search identified 2283 articles (Fig. 1). One study was not captured by the data search and was found by screening the references of an eligible study. After duplicates were removed, 1224 studies were screened using title and abstract for relevance. The remaining 67 articles were screened using the full text to assess for eligibility based on the inclusion criteria, of which 44 were excluded. Common reasons for study exclusion were as follows: studies were not healthcare related (sports, music, quality improvement rapid cycles, veterinary medicine), and studies did not have the primary features of RCDP healthcare simulation despite having elements of deliberate practice and/or mastery learning (i.e., no within event debriefing or coaching, no expert feedback, no repetition, no case progression, or no progressively challenging cases). Two study members independently performed title, abstract, and full text screening. Disagreements were settled by consensus. The included studies were then categorized based on design, country, participants, interventions, comparisons if any, and outcome variables measured. The outcomes were classified based on Kirkpatrick’s model for training program evaluation: K1, reaction (the learner’s reaction to the activity); K2, learning (objective measures of knowledge and skills acquired); K3, behavior (application of what was learned in the clinical real-life environment); and K4, results (systemic outcomes such as improved patient outcomes, cost-savings, etc.) [8]. Given the heterogeneity of the studies and the broad scope of this review, no critical appraisal of the evidence was performed.

## Results

This review included 23 studies [5, 9–30]. Table 1 outlines study characteristics, intervention, comparison, and outcomes. Nineteen studies were from the USA. Study designs were diverse; the most common study design was pre-/post-test (10 studies), and only five studies were randomized controlled studies. Most participants were residents (8 studies) with pediatric residents being most common (5 studies). The most common type of simulation scenarios that RCDP was used for was pediatric resuscitation (5 studies), pediatric critical care (4 studies), and adult resuscitation (4 studies). Of the 23 studies, 15 studies directly assessed RCDP, while the remaining 8 studies had RCDP as part of mixed interventions. For example, Brown et al. studied a 1-day bootcamp where two RCDP scenarios were part of a larger intervention involving didactic lectures, case studies, and traditional simulation [12]. Most studies did not have a comparison; five studies compared RCDP to traditional simulation, two studies to standard training, one study to different training frequencies and standard curriculum, and one study to previous traditional simulation experience.

**Table 1:** Description of included studies

**Study, design, and country**	**Participants**	**Type of simulation**	**Intervention**	**Comparison**	**Outcome measures**	**Results** (no K4 outcomes reported)		
						**K1**	**K2**	**K3**
Ahmed et al., 2020 Pre-/post-intervention pilot curriculum, USA	Emergency medicine residents (*n* = 22)	Death notification	RCDP	None	Survey, MCQ, critical action checklist (death notification score)	Significant increase in median self-efficacy/confidence survey scores	Significant increase in MCQ, death notification scores	
Bordelon et al*.,* 2020 Descriptive, USA	Neonatal nurse practitioner students (*n* = 20)	Neonatal abstinence syndrome (nontechnical)	RCDP	None	Post-simulation evaluation	Self-reported increased organization and confidence in caring for infants with NAS, heightened empathy for mother-infant dyad, increased confidence with family communication		
Brown et al*.*, 2018 Prospective pre-/post-intervention pilot study, USA	Acute care pediatric nurse practitioners (*n* = 30)	Pediatric critical care (complex post-operative congenital heart disease complications)	Mixed (didactic lectures, case studies, traditional simulation, RCDP)	None	MCQ, time-to-task, checklist, student satisfaction and self-confidence in learning tool	High level of satisfaction and confidence	Overall median time-to-treat improved Significant increase in clinically time-sensitive tasks completed in 5 min Significant decrease in time-to-task for pulmonary hypertension scenario. No significant difference in overcirculated Norwood/BT shunt scenario	
Brown et al*.*, 2020 Prospective pre-/post-intervention study, USA	Acute care pediatric nurse practitioners (*n* = 25)	Pediatric resuscitation, pediatric critical care emergencies	Mixed (didactic lectures, procedural task training, traditional simulation, RCDP)	None	MCQ, time-to-task, 3-month follow-up survey	100% felt prepared to lead a pediatric intensive care emergency in a 3 month follow up survey	Significant increase in MCQ Significant improvement in resuscitation variables Significant improvement to defibrillate within 180 s	-
Chancey et al*.*, 2018 Qualitative study, USA	Pediatric emergency nurses and residents (*n* = 44)	Pediatric resuscitation	RCDP	Past experience with traditional simulation	Surveys and interviews	RCDP well received by learners 3 main themes: real-time corrections allowed for repetition and practice; increased confidence; “smaller chunks” maximized learning without cognitive overload RCDP vs past history of sim: more focused error correction, skill acquisition, and practice		-
Colman et al*.*, 2019 Pre-/post-intervention simulation-based team training study, USA	Nurses, respiratory therapists (*n* = 76)	Pediatric rapid response team nontechnical skills training	RCDP	None	Clinical training scale (CTS) tool, pre-/post-intervention survey of floor staff, 6-month learner follow-up survey	RCDP “well perceived” in 6 month learner follow-up survey	CTS mean scores improved from poor to average/good after 1st scenario	Floor staff survey: perceived improvement in leadership, role identification, communication, and flattened hierarchy
Cory et al*.*, 2019 Prospective, randomized control study, USA	Pediatric residents (*n* = 46)	Pediatric sepsis	RCDP Traditional simulation	Traditional simulation	MCQ, checklist, time-to-task, 3–4-month follow-up MCQ and checklist		Both groups increased MCQ and checklist score post-intervention RCDP vs traditional sim: No difference in MCQ scores b/w groups. Significantly higher checklist score in RCDP group Time to task: both decreased. RCDP significantly faster for time to first bolus 3 − 4-month follow-up: no change in MCQ, checklist scores lower for both groups vs immediate post-intervention. RCDP significantly greater change in checklist score from pre-intervention to follow-up	
Gross et al*.*, 2019 Randomized controlled single-blinded study, USA	Medical students, pharmacy students (*n* = 35)	Pediatric intubation procedure	RCDP Traditional simulation	Traditional simulation	Checklist		RCDP vs traditional sim: RCDP group had significantly higher overall checklist score change post-intervention No significant difference in mean difference between both groups for endotracheal tube placement success	
Gupta et al*.*, 2019 Descriptive (program pilot report), Canada	Ward nurses, ward physicians and residents (*n* = 37)	Pediatric resuscitation (first five minutes of cardiac arrest before arrival of code team)	Mixed (procedural task training, RCDP in situ simulation)	None	Survey	Pilot program rated as “very useful” or “extremely useful”		
Hunt et al*.*, 2014 Prospective pre-/post-interventional study, USA	Pediatric residents (*n* = 72 in pre-intervention, *n* = 51 post-intervention). 2 cohorts separated by 2 years	Pediatric resuscitation	RCDP	Standard curriculum (no RCDP). Historical control	Time-to-task		RCDP (“post-intervention”) group, significant decrease in no-flow fraction (proportion of time arrest patient received no chest compressions), no-blow fraction (proportion of time arrest patient received no BVM), median time from onset of pulseless vtach to defibrillation, pre-shock pause vs. pre-intervention group	
Hunt et al*.*, 2017 Prospective randomized controlled study, USA	Medical students (*n* = 122)	Basic life support (BLS)	“HospBLS” curriculum (BLS + in-hospital skills + RCDP)	Standard BLS (“TradBLS”)	Chest compression fraction (CCF), time-to-task, checklist		HospBLS significantly larger CCF and faster median time to compressions vs TradBLS for in- and out-of- hospital arrest No significant difference in time-to-defibrillation (both within 180 s) HospBLS performed more hospital-specific maneuvers to optimize compressions and airway in in-hospital arrest scenario	
Jeffers and Poling 2019 Descriptive, USA	Pediatric emergency medicine fellows *(n* = 8)	Pediatric emergency care	Mixed (procedural skills training, traditional simulation, RCDP)	None	Survey, focus group interview, debriefing assessment for simulation in healthcare (DASH)	High learner satisfaction and perceived educational value	DASH: learners rated instructor quality highly. Lowest rated domain was organization of instructor’s debrief	
Kosoko et al*.,* 2019 Pre-test and post-test course study, Botswana	Prehospital nurses and emergency medical technician (*n* = 31)	Prehospital stabilization and resuscitation	Mixed (didactic lectures, procedural skills training, RCDP)	None	MCQ, checklist, survey	Significant increase in median self-efficacy scores All participants found the course useful	Increased MCQ, simulation based checklist scores from pre- to post-test	
Kutzin and Janicke 2015 Descriptive, USA	Nurses (*n* = not reported)	Resuscitation (first five minutes before code team arrives)	RCDP	None	Clinical reports	Participants report greater retention of first five minutes skills and high learner satisfaction		Clinical reports from hospital: nursing staff better able to manage cardiac arrest, faster initiation of compressions, better patient positioning, and necessary arrest equipment in room
Lemke et al*.*, 2016 Prospective pre-test and post-test pilot study, USA	Pediatric emergency medicine fellows, nurses, respiratory therapists (*n* = 22, only 7 were present on both training days)	Pediatric resuscitation	RCDP Traditional simulation	Traditional simulation	Simulation team assessment tool (STAT)	Fellows who were present on both training days liked RCDP more, but greater fatigue with RCDP	Significant improvement overall STAT with RCDP, no significant improvement with traditional simulation RCDP vs traditional simulation: significant difference in STAT human factors sub-section (RCDP improved 10.2%, traditional sim 1.7%). No significant difference in overall STAT score b/w 2 groups	
Magee et al., 2018 Prospective, randomized control study, USA	Pediatric interns (*n* = 34)	Neonatal resuscitation	RCDP Traditional simulation	Traditional simulation (“SD”)	Megacode assessment form (MCAF), time-to-task, survey, 4-month MCAF	Increased confidence for both groups	Immediate: RCDP MCAF overall score significantly higher than SD. No difference in harmful actions performed between 2 groups Time-to-task for PPV and epinephrine administration significantly faster in RCDP 4-month follow-up: recall MCAF not significantly different b/w 2 groups Decrease in score from immediate to recall session greater in RCDP than SD	
McPhee 2018 Descriptive, USA	Nurse residents (*n* = 135)	Resuscitation (mock codes)	RCDP	None	Learner evaluation	High satisfaction due to feedback, skill repetition, and the opportunity to fix errors on subsequent cycles 100% reported increased knowledge and satisfaction 98% felt sim experience covered critical content		
Patricia et al*.*, 2017 Descriptive, USA	Physicians, nurses, respiratory therapists (*n* = 128)	Neonatal Resuscitation	RCDP	None	Anecdotal	Increased confidence High satisfaction		
Powers et al*.*, 2018 Descriptive, USA	Nursing students	ACLS	Mixed (procedural skills training, RCDP sim)	None	Anecdotal reports	Learners found RCDP “Effective in teaching ACLS skills”, and increased confidence		
Rosman et al*.*, 2019 Pre-test and post-test curriculum study, Rwanda	Pediatric residents (*n* = 33) Enrolled 51 but due to technical issues in low resource setting, only 33 included	Pediatric emergency care in low resource setting	RCDP Traditional simulation	Traditional simulation	STAT, surveys	Significant increase in self-confidence from pre- to post-testing Trend towards greater improvement in self confidence in RCDP vs traditional, but no significant difference between RCDP and traditional group	Significant increase in STAT scores from pre- to post- test in both groups No significant difference in percent change in STAT score between RCDP and traditional groups	
Sullivan et al*.*, 2015 Randomized control study, USA	Nurses (*n* = 66)	Resuscitation/in-hospital cardiac arrest (ICHA) prior to code team arrival	In situ RCDP	Training every 2 months vs 3 months vs 6 months vs control (standard AHA training)	Time-to-task, CCF		Significant decrease in median time-to-task for initiation of chest compressions and defibrillation b/w 2 mo, 3 mo vs control. No significant difference between 6 mo group vs control CCF increased as time between training intervals decreased. Significant difference b/w 2 mo and 3 mo groups vs control, but not for 6mo group vs control	
Yan et al*.*, 2020 Pre-/post-intervention curriculum study, USA	Surgical interns (*n* = 16)	Pediatric trauma primary and secondary survey	RCDP	None	Survey	Significant improvement in self-confidence from pre to post High learner satisfaction		
Zern et al*.*, 2020 Pre-test and post-test curriculum study, USA	Internal medicine and family medicine residents (*n* = 27)	Resuscitation (ACLS and teamSTEPPS teamwork and communication)	In situ simulation for needs assessment × 5. Curriculum developed. Then: mixed (didactic, RCDP sim)	None	Checklist, time-to-defibrillation		Significant improvement in cardiac arrest team leader performance checklist and time to defib from pre to post test	

*RCDP* rapid cycle deliberate practice, *MCQ* multiple choice question, *BLS* basic life support, *ACLS* advanced cardiac life support, *CCF* chest compression fraction, *STAT* simulation team assessment tool

All studies used a similar definition of RCDP, as introduced by Hunt et al. in 2014, and used variations of pausing, micro-debriefing or coaching, and rewinding [5]. However, there were differences in how the RCDP technique was implemented as outlined in Table 2. Many studies had the first simulation run without interruption to act as a needs assessment. Studies with pre-determined pauses split the cases into smaller segments or learning chunks purposely, while other studies paused for praise or error correction. During the pauses or completion of the short cases, micro-debriefing, feedback, or coaching was completed in all studies. After the pause, rewinds occurred to either the beginning and/or to just before the pause. Most cases progressed, and 7 studies reported progressive difficulty. For example, Lemke et al. had medical objectives sequenced into progressively more difficult rounds [22]. Each subsequent round was built on previously mastered skills and escalated in difficulty. The supraventricular tachycardia case started with basic communication objectives, and the subsequent rounds required cardioversion and cardiac arrest management [22].

**Table 2:** Characteristics of RCDP implementation

Technique	Number of studies	Studies
1st simulation uninterrupted	11	Ahmed et al*.,* 2020, Brown et al*.,* 2018, Chancey et al*.,* 2018, Colman et al*.,* 2019, Cory et al*.,* 2019, Gross et al*.,* 2019, Hunt et al*.,* 2014, Lemke et al*.,* 2016, Magee et al*.,* 2018, Sullivan et al*.,* 2015, Zern et al*.,* 2020
Pre-determined pauses	9	Bordelon et al*.,* 2020, Brown et al*.,* 2018, Colman et al*.,* 2019, Cory et al. 2019, Gross et al*.,* 2019, Kutzin and Janicke 2015, McPhee 2018, Rosman et al*.,* 2019, Zern et al*.,* 2020
Pauses for error correction/praise/feedback	10	Ahmed et al*.,* 2020, Chancey et al*.,* 2018, Colman et al*.,* 2019, Cory et al*.,* 2019, Gross et al*.,* 2019, Hunt et al*.,* 2014, Lemke et al*.,* 2016, Magee et al*.,* 2018, Yan et al*.,* 2020, Zern et al*.,* 2020
Short cases repeated multiple times	2	Gupta et al*.,* 2019, Sullivan et al*.,* 2015
Rewind to start	9	Brown et al*.,* 2018, Cory et al*.,* 2019, Gupta et al*.,* 2019, Magee et al*.,* 2018, Kutzin and Janicke 2015, Lemke et al*.,* 2016, McPhee 2018, Rosman et al*.,* 2019, Sullivan et al*.,* 2015
Rewind to pause	7	Chancey et al*.,* 2018, Colman et al*.,* 2019, Cory et al*.,* 2019, Gross et al*.,* 2019, Hunt et al*.,* 2014, Lemke et al*.,* 2016, Yan et al*.,* 2020
Case progression	15	Ahmed et al*.,* 2020, Bordelon et al*.,* 2020, Brown et al*.* 2018, Chancey et al*.,* 2018, Colman et al*.,* 2019, Cory et al. 2019, Gross et al*.,* 2019, Hunt et al*.,* 2014, Lemke et al*.,* 2016, Magee et al*.,* 2018, Kutzin and Janicke 2015, McPhee 2018, Rosman et al*.,* 2019, Yan et al*.,* 2020, Zern et al*.,* 2020
Increasing difficulty	6	Bordelon et al*.,* 2020, Chancey et al*.,* 2018, Hunt et al*.,* 2014, Lemke et al*.,* 2016, Rosman et al*.,* 2019, Yan et al*.,* 2020
Incomplete information regarding implementation	5	Brown et al*.,* 2020, Hunt et al*.,* 2017, Jeffers and Poling 2019, Kosoko et al*.,* 2019, Powers et al*.,* 2018. Note: Brown et al., 2020 and Hunt et al. 2017 had previous studies that did list their protocol

Outcome measures and results are listed in Table 1. The most common K1 outcomes were increased learner confidence (11 studies) and learner satisfaction (11 studies) in RCDP alone and with mixed interventions. At the K2 level, RCDP was associated with knowledge and skills acquisition demonstrated by significantly increased multiple choice question (MCQ) scores (4 studies), increased checklist scores (8 studies), decreased time to critical tasks (6 studies), and increased chest compression fraction (CCF) (2 studies). For example, a 2-day pre-hospital care provider course in a low resource setting consisted of didactic lectures, skills training, and multiple RCDP simulations. All participants found the course “useful”, and it resulted in significant increases in MCQ scores (pretest 67%, post-test 85%, *p* < 0.001) and simulation checklist performance scores (pretest 42%, post-test 95%, *p* < 0.001) [20]. At the K3 level, a floor staff survey found that RCDP participants had perceived improvement in leadership, communication, and role identification [14]. Additionally, hospital clinical reports found that nursing staff were better able to manage cardiac arrest after RCDP training [21]. No K4 outcomes were reported.

When comparing interventions with RCDP to standard curriculums, RCDP was associated with a significant decrease in time to tasks, such as the proportion of time a cardiac arrest patient received no chest compressions (no-flow fraction), time to defibrillation, and an impressive tenfold reduction in pre-shock pause [5]. A significantly larger CCF and decreased time to compression initiation were also found when examining RCDP compared to traditional BLS training [18]. Interestingly, this study did not find a significant difference in decreased time to defibrillation between the 2 groups, but defibrillation still occurred within the American Heart Association (AHA) guidelines of 180 s [18]. Sullivan et al. examined outcomes with different training frequencies and found that RCDP training for cardiac arrest skills at 2- and 3-month intervals were associated with significant decreased time to initiation of chest compressions and defibrillation when compared to 6-month intervals and standard AHA curriculum [28]. The authors concluded that the ideal time for RCDP resuscitation skills training is every 3 months [28].

When comparing RCDP to traditional simulation, the results were mixed. RCDP participants commented on liking RCDP more [13, 22], but feeling more fatigued [22]. Rosman et al. did not find a significant difference in self-confidence between the 2 groups [27]. The results were also mixed at the K2 level. Although RCDP was associated with decreased times to critical tasks [15, 23], significantly higher checklist scores [15, 16, 23], and significantly higher simulation team assessment tool (STAT) human factors sub-section scores [22], there was no significant difference in overall STAT score improvement [22, 27], and no difference in MCQ scores [15] between the two groups. When examining 3–4-month recall, checklist scores decreased in both groups with the RCDP groups having greater decreases in scores compared to the traditional simulation groups [15, 23].

## Discussion

To the best of our knowledge, this is the largest review to date that examines and summarizes the RCDP literature. This scoping review consists of 23 published studies, 21 more than the previous review [7]. The studies were heterogeneous in design, participants, simulation types, and outcome measures. Implementation strategies also varied. Interestingly, all studies referenced Hunt et al. for their definitions of RCDP [5]. They included the core techniques of RCDP, including within-event debriefing, deliberate practice, clear learning objectives, formative assessment, and pause-feedback-replay loops. However, the variations in the use of a needs assessment, pause timing, rewinding or restarting, case progression, and escalating difficulty make it difficult to draw general conclusions about RCDP. As RCDP becomes more popular, it is important that implementation and simulation design become standardized to better research its effects.

This review found that RCDP training is associated with multiple positive outcomes including learner satisfaction, increased confidence, knowledge and skills acquisition, and time to critical tasks. RCDP allows learners to receive timely direct feedback and multiple opportunities to “do it right”; they can incorporate feedback and try again immediately. Breaking cases down into “smaller chunks” maximizes learning without cognitive overload [13]. This approach likely works well for skills that are algorithmic, have defined protocols, or clear scripts. Although all studies reported positive K1/K2 outcomes, most studies did not compare RCDP to other interventions so their conclusions may be from the education itself or from the instruction technique. However, the K2 outcomes demonstrated, such as the direct observation of key tasks, may contribute to the overall competency of the learner [31]. Despite the small numbers of randomized controlled studies and lack of intervention comparisons, RCDP should still be considered useful in medical education. Furthermore, while the previous review only had studies that examined technical resuscitation or procedural skills, this review includes studies that looked exclusively at nontechnical skills including death notification and family communication [7, 9, 10]. This implies that RCDP simulation can be successfully employed to teach nontechnical abilities. Since there is limited research on the impact of RCDP training outside of the learning environment, future studies should examine how RCDP interventions translate to clinical practice.

This review examined comparisons between interventions with RCDP to standard curriculums and traditional simulation. It appears that adding RCDP to standard curriculums is overall beneficial, leading to multiple improvements in K2 outcomes. However, when compared to traditional simulation, the results are mixed. While some studies showed immediate improvements, three of the five studies that compared RCDP to traditional simulation did not show significant differences in all K2 outcome measures [15, 22, 27]. Furthermore, RCDP groups had greater decreases in scores at 3–4-month follow-ups compared to the traditional simulation groups even though these studies both showed significantly higher scores in the RCDP groups immediately after training [15, 23]. This suggests that although RCDP has an immediate advantage in knowledge application, knowledge retention has not been demonstrated. Despite being able to apply feedback immediately with repetition to reinforce learned skills, it is possible that long-term “muscle memory” is not being created with RCDP. One study recommended that RCDP training should occur every 3 months to maintain skills and avoid skills decay observed at 6 months [28]. More studies are needed to compare RCDP to other types of SBME, and specifically how skills retention with RCDP compares with other modes of instruction. This review has not found that RCDP is superior to traditional simulation.

There are limitations to this review. Grey literature was not searched, and conference abstracts and poster presentations were excluded. Studies were also limited to the English language. Critical appraisal of study quality was not completed. Most studies were small and at a single site. Many of the studies had no comparisons, and the validity and reliability of the outcome measurement tools were not well described. It is possible that their outcomes may be related to other confounders.

## Conclusion

RCDP has rapidly emerged since it was introduced by Hunt et al. in 2014 [5]. The definition of RCDP is consistent across the literature, but implementation strategies vary. The current literature suggests that RCDP technical and nontechnical skills training are associated with positive outcomes in learner performance. It is likely that adding RCDP to standard curriculums is overall beneficial, but unclear if RCDP is superior to traditional simulation. More studies are needed to examine how RCDP translates to clinical practice, long-term skills retention with RCDP training, and comparisons of RCDP training to other types of instruction.

## Appendix I Database Search strategies

Ovid MEDLINE(R) ALL < 1946 to October 12, 2020 > 

Search history sorted by search number ascending

**Table:**

**#**	**Searches**	**Results**	**Type**
1	RCDP.mp	127	Advanced
2	Deliberate practice.mp	723	Advanced
3	(Rapid cycle adj3 feedback).mp	16	Advanced
4	(Rapid cycle adj3 feedback).mp	0	Advanced
5	(Rapid cycle adj3 practice*).mp	33	Advanced
6	or/1–5	850	Advanced
7	Rhizomelic chondrodysplasia punctata.mp	254	Advanced
8	6 not 7	757	Advanced
9	Remove duplicates from 8	688	Advanced
10	Limit 9 to English language	684	Advanced

Embase < 1974 to 2020 October 12 > 

Search history sorted by search number ascending

**Table:**

**#**	**Searches**	**Results**	**Type**
1	RCDP.mp	155	Advanced
2	Deliberate practice.mp	903	Advanced
3	(Rapid cycle adj3 feedback).mp	30	Advanced
4	(Rapid cycle adj3 feedback).mp	0	Advanced
5	(Rapid cycle adj3 practice*).mp	48	Advanced
6	or/1–5	1071	Advanced
7	Rhizomelic chondrodysplasia punctata.mp	289	Advanced
8	6 not 7	964	Advanced
9	Remove duplicates from 8	945	Advanced
10	Limit 9 to English language	939	Advanced
11	Limit 10 to (books or chapter or conference abstract or conference paper or "conference review" or book or book series or conference proceeding)	325	Advanced
12	10 not 11	614	Advanced

APA PsycInfo < 1806 to October week 1 2020 > 

Search history sorted by search number ascending

**Table:**

**#**	**Searches**	**Results**	**Type**
1	RCDP.mp	0	Advanced
2	Deliberate practice.mp	545	Advanced
3	(Rapid cycle adj3 feedback).mp	3	Advanced
4	(Rapid cycle adj3 feedback).mp	0	Advanced
5	(Rapid cycle adj3 practice*).mp	0	Advanced
6	or/1–5	548	Advanced
7	Rhizomelic chondrodysplasia punctata.mp	3	Advanced
8	6 not 7	548	Advanced
9	Remove duplicates from 8	548	Advanced
10	Limit 9 to English language	523	Advanced

Cochrane central register of controlled trials < 2014 to present > 

Search history sorted by search number ascending

**Table:**

**#**	**Searches**	**Results**	**Type**
1	RCDP.mp	7	Advanced
2	Deliberate practice.mp	114	Advanced
3	(Rapid cycle adj3 feedback).mp	2	Advanced
4	(Rapid cycle adj3 feedback).mp	0	Advanced
5	(Rapid cycle adj3 practice*).mp	12	Advanced
6	or/1–5	117	Advanced
7	Rhizomelic chondrodysplasia punctata.mp	0	Advanced
8	6 not 7	117	Advanced
9	Remove duplicates from 8	110	Advanced

Cochrane database of systematic reviews < 2005 to present > 

Search history sorted by search number ascending

**Table:**

**#**	**Searches**	**Results**	**Type**
1	RCDP.ti,ab	0	Advanced
2	Deliberate practice.ti,ab	0	Advanced
3	(Rapid cycle adj3 feedback).ti,ab	0	Advanced
4	(Rapid cycle adj3 feedback).ti,ab	0	Advanced
5	(Rapid cycle adj3 practice*).ti,ab	0	Advanced
6	or/1–5	0	Advanced
7	Rhizomelic chondrodysplasia punctata.ti,ab	0	Advanced
8	6 not 7	0	Advanced

**Table:**

**#**	**Query**	**Limiters/expanders**	**Last run via**	**Results**
S6	S1 OR S2 OR S3 OR S4 OR S5	Expanders—Apply equivalent subjects Search modes—Boolean/Phrase	Interface—EBSCOhost Research Databases Search Screen—Advanced Search Database—CINAHL with Full Text	353
S5	TI (rapid cycle N3 practice*) OR AB (rapid cycle N3 practice*)	Expanders—Apply equivalent subjects Search modes—Boolean/Phrase	Interface—EBSCOhost Research Databases Search Screen—Advanced Search Database—CINAHL with Full Text	21
S4	TI (rapid cycle N3 feedback) OR AB (rapid cycle N3 feedback)	Expanders—Apply equivalent subjects Search modes—Boolean/Phrase	Interface—EBSCOhost Research Databases Search Screen—Advanced Search Database—CINAHL with Full Text	0
S3	TI (rapid cycle N3 feedback) OR AB (rapid cycle N3 feedback)	Expanders—Apply equivalent subjects Search modes—Boolean/Phrase	Interface—EBSCOhost Research Databases Search Screen—Advanced Search Database—CINAHL with Full Text	8
S2	TI deliberate practice OR AB deliberate practice	Expanders—Apply equivalent subjects Search modes—Boolean/Phrase	Interface—EBSCOhost Research Databases Search Screen—Advanced Search Database—CINAHL with Full Text	335
S1	TI RCDP OR AB RCDP	Expanders—Apply equivalent subjects Search modes—Boolean/Phrase	Interface—EBSCOhost Research Databases Search Screen—Advanced Search Database—CINAHL with Full Text	11
